# Sente-se Menos, Mova-se Mais e Sinta-se Bem, Pessoal!: O Comportamento Sedentário pode Comprometer a Saúde Cardiometabólica por Meio de Problemas de Saúde Mental ao Longo da Vida

**DOI:** 10.36660/abc.20220894

**Published:** 2023-02-13

**Authors:** Ceren Bicer, Yasin Hasan Balcioglu

**Affiliations:** 1 Hacettepe University Faculty of Medicine Ankara Turquia Hacettepe University Faculty of Medicine, Ankara – Turquia; 2 Department of Psychiatry Research Hospital for Psychiatry, Neurology, and Neurosurgery Istanbul Turquia Department of Psychiatry, Bakirkoy Prof Mazhar Osman Training and Research Hospital for Psychiatry, Neurology, and Neurosurgery, Istanbul – Turquia

**Keywords:** Risco Cardiovascular, Adolescência, Idade Adulta, Hábitos Alimentares, Doença Psiquiátrica

Lemos com interesse o artigo intitulado “Comportamento sedentário, hábitos alimentares e risco cardiometabólico em crianças e adolescentes fisicamente ativos”.^1^ Neste estudo, os autores tentaram avaliar a relação entre comportamento sedentário, fatores de risco cardiometabólico e hábitos alimentares em crianças e adolescentes fisicamente ativos. Seus resultados concluíram que não há associação entre comportamento sedentário e fatores de risco cardiometabólicos em crianças e adolescentes fisicamente ativos. No entanto, concluíram que o comportamento sedentário estava associado a hábitos alimentares inadequados. Além disso, crianças e adolescentes com comportamento sedentário apresentaram maior probabilidade de consumir alimentos em frente à televisão regularmente e consumir pelo menos um alimento ultraprocessado por dia, consumindo menos frutas.

Apesar deste estudo fornecer achados importantes, os estudos com desenho transversal têm inerentemente limitações substanciais. Particularmente quando se trata de doenças como distúrbios cardiometabólicos, estes são os resultados da acumulação de fatores de risco ao longo de muitos anos. Um delineamento transversal para essa faixa etária pode negligenciar os efeitos do comportamento sedentário nas condições cardiometabólicas na idade adulta, pois provavelmente não terá tempo suficiente para demonstrar seu real efeito no início da vida.

Em segundo lugar, um estilo de vida sedentário é um problema de saúde pública emergente nas sociedades desenvolvidas e em desenvolvimento. Níveis mais elevados de comportamento sedentário são um conhecido fator de risco para ganho de peso, adiposidade abdominal e doenças cardiometabólicas em crianças, adolescentes e adultos.^2 - 5^ O que também é amplamente reconhecido são os efeitos positivos da atividade física na saúde mental, mas pouco se sabe sobre os efeitos dos comportamentos sedentários na saúde mental.^6^ O presente estudo indicou que o comportamento sedentário está relacionado com hábitos alimentares pouco saudáveis. Assim, os efeitos do comportamento sedentário na saúde mental também devem ser mencionados. Uma revisão sistemática de nove estudos transversais encontrou uma relação negativa entre autoestima e comportamento sedentário e uma correlação positiva entre estilo de vida sedentário e transtornos alimentares.^7^ Estudos recentes também indicaram uma associação entre comportamentos sedentários, depressão e aumento do risco de suicídio em populações de adolescentes e adultos.^8 - 10^ Um estilo de vida sedentário também está associado a interrupções do ritmo circadiano, e o comprometimento das características cronobiológicas está associado a um risco aumentado de doenças cardiometabólicas, principalmente em pacientes psiquiátricos.^11 , 12^

Nosso conhecimento sobre os efeitos de um estilo de vida sedentário em transtornos alimentares e escolhas de consumo ainda é limitado, embora o estudo atual e outros tenham indicado hábitos alimentares alterados e disfuncionais que predispõem a adversidades cardiometabólicas. Por outro lado, é possível afirmar que um estilo de vida sedentário tem efeitos adversos de longo prazo sobre a saúde mental e leva a muitos transtornos psiquiátricos, que estão relacionados à morbidade não apenas por causa da carga de saúde mental, mas são fatores predisponentes bem conhecidos fatores para doenças cardiometabólicas. Estudos prospectivos são necessários para esclarecer o papel de um estilo de vida sedentário em idades precoces no desenvolvimento de adversidades cardiometabólicas na idade adulta na moderação de transtornos psiquiátricos.
