# Supplementary material for: Sente-se Menos, Mova-se Mais e Sinta-se Bem, Pessoal!: O Comportamento Sedentário pode Comprometer a Saúde Cardiometabólica por Meio de Problemas de Saúde Mental ao Longo da Vida
Source: Arq Bras Cardiol. 2023 Feb 13;120(2):e20220894. [Article in Portuguese] doi: 10.36660/abc.20220894 (PMC9972661; doi:10.36660/abc.20220894)
Supplement: Translation

# Translation: Sit Less, Move More, and Feel Good, Folks!: Sedentary Behavior May Stake Cardiometabolic Health Through Mental Health Problems Across the Lifespan

**Authors:** Ceren Bicer, Yasin Hasan Balcioglu

**Affiliations:** 1 Turquia Hacettepe University Faculty of Medicine, Ankara – Turquia; 2 Turquia Department of Psychiatry, Bakirkoy Prof Mazhar Osman Training and Research Hospital for Psychiatry, Neurology, and Neurosurgery, Istanbul – Turquia

**Keywords:** Cardiovascular Risk, Adolescence, Adulthood, Eating Habits, Psychiatric Illness

We read with interest the article titled “Sedentary behavior, dietary habits and cardiometabolic risk in physically active children and adolescents”.^1^In this study, the authors attempted to evaluate the relationship between sedentary behavior, cardiometabolic risk factors, and eating habits in physically active children and adolescents. Their results concluded that there is no association between sedentary behavior and cardiometabolic risk factors in physically active children and adolescents. However, they concluded that sedentary behavior was associated with inappropriate eating habits. Moreover, children and adolescents showing sedentary behavior were more likely to consume food in front of the television regularly and to consume at least one ultra-processed food per day while consuming less fruit. We want to address several issues in this valuable article.

Despite this study providing important findings, studies with cross-sectional design inherently have substantial limitations. Particularly when it comes to diseases such as cardiometabolic disorders, these are the results of the cumulation of risk factors over many years. A cross-sectional design for this age group may overlook the effects of sedentary behavior on cardiometabolic conditions in adulthood, as it most likely will not have enough time to demonstrate its real effect during early life.

Secondly, a sedentary lifestyle is an up-and-coming public health problem in developing and developed societies. Higher levels of sedentary behavior are a known risk factor for weight gain, abdominal adiposity, and cardiometabolic diseases in children, adolescents, and adults.^2 - 5^What is also widely recognized that the positive effects of physical activity on mental health, but less is known about the effects of sedentary behaviors on mental health.^6^The current study indicated that sedentary behavior is linked with unhealthy eating habits. Thus, the effects of sedentary behavior on mental health should also be mentioned. A systematic review of nine cross-sectional studies found a negative relationship between self-esteem and sedentary behavior and a positive correlation between a sedentary lifestyle and eating disorders.^7^Recent studies also indicated an association between sedentary behaviors, depression, and increased risk of suicide in both adolescent and adult populations.^8 - 10^A sedentary lifestyle is also associated with circadian rhythm disruptions, and impairment in chronobiological features is linked with an increased risk of cardiometabolic diseases, particularly in psychiatric patients.^11 , 12^

Our knowledge about the effects of a sedentary lifestyle on eating disorders and consumption choices is still limited, although the current study and others indicated altered and dysfunctional eating habits predisposing to cardiometabolic adversities. On the other hand, it is possible to claim that a sedentary lifestyle has long-term adverse effects on mental health and leads to many psychiatric disorders, which are related to morbidity not only because of mental health burden, but they are well-known predisposing factors for cardiometabolic diseases. Prospective studies are warranted to enlighten the role of a sedentary lifestyle at early ages in the development of adulthood cardiometabolic adversities in the moderation of psychiatric disorders.
